# Research on the factors and path of sports industry development in China: A fsQCA study of 31 provinces

**DOI:** 10.1371/journal.pone.0298167

**Published:** 2024-04-16

**Authors:** Yi Shi, Qingyao Song, Lin Li

**Affiliations:** 1 School of Sports Training, Chengdu Sport University, Chengdu, Sichuan, China; 2 Sports Department of Jiangsu University, Zhenjiang, Jiangsu, China; Harbin Institute of Technology, CHINA

## Abstract

The advancement of the sports industry’s development constitutes a critical concern shared by regional authorities and the scholarly community, reflecting its significant role in economic and social development. This study employs a Fuzzy-set Qualitative Comparative Analysis (fsQCA) methodology to examine the 31 provincial-level administrative units in China. The objective is to elucidate the influence of technological, organizational, and environmental factors on the industry’s development level, considering both a holistic national framework and dissected regional approaches (Eastern, Central, and Western China). This paper’s contribution to the literature is structured around the following core findings: (1) The study establishes that a singular condition does not suffice as an essential prerequisite for achieving a heightened development state within the sports industry. (2) At the national level, there are three pathways to enhance the development level of the sports industry, specifically identified as "network-human resources dominant pathway," "technological innovation-human resources dominant pathway," and "comprehensive synergistic pathway."(3) From a regional perspective, the Eastern region has two pathways for sports industry enhancement: "network-economic pathway" and "comprehensive synergistic pathway." The Central region follows a "technology pathway," while the Western region has three pathways: "organization-environment pathway," "network-organization-environment pathway," and "organization pathway."(4) The synthesis of these findings underscores the multifactorial nature of sports industry development, suggesting a paradigm where diverse routes can lead to equivalent outcomes. This heterogeneity indicates that provinces or regions can tailor their development strategies to their unique situational contexts.

## 1. Introduction

Since the issuance and implementation of the State Council’s "Several Opinions on Accelerating the Development of the Sports Industry and Promoting Sports Consumption," the sports industry has witnessed an accelerated trajectory of growth. The policy catalyzed significant advancements throughout the "Thirteenth Five-Year Plan," culminating in the vitalization of the sports market.

On August 10, 2019, the General Office of the State Council promulgated the Outline for the Construction of a Sports Power (Guo Ban Fa [2019] No. 40) [1], a seminal document delineating the strategic goal to elevate the sports industry to a cornerstone of the national economy by the year 2035. This initiative represents an unprecedented move at the policy-making level, explicitly charting the strategic course for the sports industry’s development and endorsing its role as an impetus for the expansion of the national economy. This policy initiative not only underscores the sector’s burgeoning significance but also earmarks it as an integral component in the broader economic development agenda [2]. Drawing upon empirical data provided by the National Bureau of Statistics and the General Administration of Sport of China, it is observed that the Chinese sports industry has demonstrated noteworthy expansion between 2014 and 2020. By the end of this period, the added value generated by the sports industry escalated to 1.0735 trillion yuan, comprising 1.06% of the nation’s Gross Domestic Product (GDP). This figure marks an increase of 0.42 percentage points over the baseline year of 2014. Exhibiting an impressive compound annual growth rate (CAGR) of 17.68%, the sports sector has surfaced as a prominent leader in China’s economic landscape, outpacing several other sectors in terms of growth velocity. When compared to the tourism and cultural sectors, the sports industry’s average annual growth rate was 2.6 and 1.5 times greater, respectively. Such comparative metrics underscore the sports industry’s robust and dynamic expansion within the broader context of China’s economy. The overarching trajectory of the sports industry, as evidenced by these metrics, is indicative of a sector that is not only burgeoning but also increasingly contributing to the diversification and vitality of China’s economic structure [3]. To contextualize the scale of the United States sports industry within the global market, it is pertinent to analyze comparative data. In 2018, the added value of the US sports industry was recorded at an impressive $539.9 billion. When juxtaposed with international figures, this statistic becomes particularly noteworthy. The global sports industry’s added value for the same year amounted to approximately $1.3 trillion. This implies that the United States contribution constituted a substantial 41.5% of the worldwide sports industry’s added value. Such data highlights the predominant position of the US sports industry in the global arena and underscores its significant impact on the economic footprint of the sports sector internationally [4].

In 2018, the added value of China’s sports industry was 1.0078 trillion yuan, equivalent to approximately 152.2 billion US dollars, based on the prevailing exchange rate of 1 US dollar to 6.62 yuan. In comparison, the US sports industry was 3.5 times larger in economic output, highlighting the substantial growth potential for China’s sports sector to become a more integral part of its national economy. Despite notable progress, strategic investments and policy reforms are essential to leverage this potential and align China’s sports industry with global leaders [5].

China’s sports industry has exhibited robust growth, yet this progress has not been without its challenges. The industry faces a homogeneous development model, cost-return imbalances, and other inefficiencies, leading to a value chain characterized by "low-end lock-in." This has culminated in reduced competitiveness, efficiency deficits, structural imbalances, and disparate regional development. Consequently, despite its potential, the industry’s impact on national economic indicators, including employment and economic stimulus, remains limited [6, 7]. In conclusion, it is crucial to examine the factors that impact the industry’s development to gain valuable insights into addressing the current challenges it faces and ensuring its continued growth and progress.

Existing literature has thoroughly examined the multifaceted factors influencing the sports industry’s evolution. Key external drivers identified include the macroeconomic environment, policy frameworks, technological advancements, and the dynamics of international trade [8–10]. On the other hand, the role of internal factors, including the caliber of human resources, consumer demand trends, and the pace of technological innovation, has been scrutinized for their contributory effects on the industry’s expansion [11, 12]. Although extant research predominantly addresses the impact of isolated factors, there is a paucity of studies examining their cumulative effects. It is crucial to recognize the interdependence of these factors and the necessity for a coordinated approach to enhance the sports industry through synergistic interplay.

To address the identified issues, this study adopts the fsQCA method to explore the growth dynamics of the sports industry. It aims to answer the following research questions within the TOE (Technology, Organization, Environment) framework: (1) What are the determinants of sports industry development according to the TOE model? (2) Under what conditions does the sports industry progress? (3) How can regions tailor their sports industry growth strategies to their unique geographical characteristics?

The study introduces an innovative TOE analytical model with profound implications for the expansion of China’s sports industry. It uncovers avenues for enhancement at the national and regional levels, providing strategic insights for the establishment of a strong sports nation. The theoretical contributions of this study are significant: it not only broadens the scope of existing research by considering the collective impact of diverse factors on the development of the sports industry but also lays a robust groundwork for policy formulation aimed at elevating the industry at national and regional scales.

## 2. Theoretical model construction

### 2.1 TOE framework

**Fig 1:**
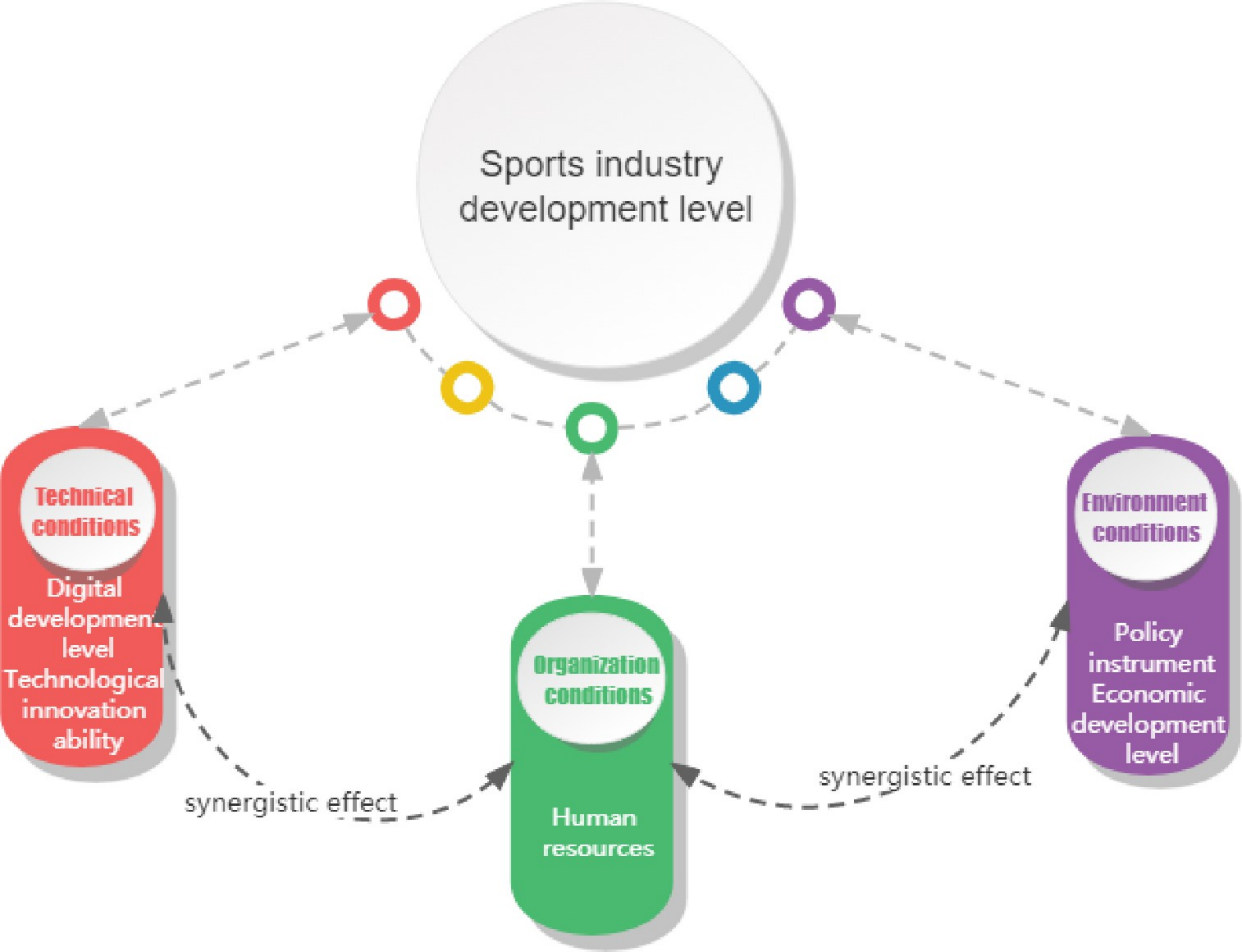
Research theoretical framework.

The TOE (Technology, Organization, Environment) framework, established by Tornatzky and Fleischer in 1990, offers a comprehensive lens for dissecting complex societal phenomena and isolating contributory factors [13]. This model serves as a robust analytical instrument for evaluating the influence of contextual factors on technological adoption outcomes, enhancing our understanding of the interactions among technology, organizational frameworks, and the environmental milieu. Its applicability transcends information systems, rendering it a versatile theoretical scaffold across disciplines such as management and sociology [14, 15]. The TOE framework stands out from other models due to its unique ability to examine the interplay of both internal and external factors. This provides a research lens that is comprehensive and far-reaching in scope. The Technology-Organization-Environment (TOE) framework has proven to be widely applicable in industrial research, spanning various sectors such as the apparel industry’s transformation [16], digitization in the cultural industry [17], and convergence dynamics in different industries [15]. This scholarly investigation focuses on the sports industry’s evolution and identifies three distinct yet interconnected dimensions: technological conditions, organizational conditions, and environmental conditions. The delineation is achieved through a rigorous analysis guided by the TOE theoretical framework. To enhance comprehension, the conceptual model underlying this research is visually represented in Fig 1.

### 2.2 Technological conditions and sports industry

The transformation of the sports industry’s developmental approach and structural makeup is attributed to the impetus of technological advancement and innovation [18]. This research identifies digitalization level and innovation capacity as principal indicators of technological determinants.

#### 2.2.1 The level of digital development and sports industry

In the context of a swiftly advancing digital landscape, the sports industry is presented with an opportunity to capitalize on digital expansion. Leveraging digital economy mechanisms can enhance resource and factor productivity [19, 20]. which may catalyze novel industrial paradigms, heightened competitiveness, and consequent economic expansion, job creation, and consumer spending. Empirical evidence suggests a positive correlation between digital maturity and enterprise resource acquisition, culminating in elevated performance [21, 22]. Consequently, a tangible association exists between digital development and the sports industry’s prosperity.

#### 2.2.2 Innovation capability and sports industry

The sports industry should prioritize innovation as the cornerstone of its developmental strategy, emphasizing the cultivation of developmental capabilities [23]. An innovation-centric paradigm is essential for structural enhancement and operational optimization within the industry [24]. Technological advancements and industry progression are accelerated by innovation. Research indicates that strategies centered on innovation are integral to economic expansion. Conversely, an innovation deficit can result in reduced economic value addition and pervasive product homogeneity within the sports sector [25].

### 2.3 Organizational conditions and sports industry

Organizational characteristics are pivotal in industrial evolution [26]. Human resources, in particular, catalyze industrial transformation, reinforce core competitiveness, and serve as strategic assets for nurturing dynamic capabilities in industrial resources [27]. thereby expanding industrial scale and advancing high-quality development. Studies underscore the critical importance of human resources in the sports industry [28], with their scarcity posing a significant barrier to the industry’s high-quality progression [29]. This highlights the indispensable connection between human resources and the sports industry’s success.**2.4 Environmental conditions and sports industry**

The trajectory of the sports industry’s development is substantially shaped by external elements such as government patronage and macroeconomic conditions. Enhanced governmental backing can catalyze market incentives, regional resource distribution, and the creation of nurturing environments for sports enterprises. Moreover, economic variables are instrumental in stimulating industry growth. This study, therefore, earmarks policy instruments and economic development indices as critical environmental indicators.

#### 2.4.1 Policy tools and sports industry

Industrial success is intricately linked to governmental engagement and direction. Governmental provision of policy frameworks and incentives is instrumental in nurturing sports industrial growth [30]. Such policies are imperative for the vigorous expansion of the sports industry by fortifying the safeguarding of industry achievements and ensuring a stable and efficient industrial operation [31]. Empirical evidence suggests that policy support is indispensable for the high-caliber development of the sports industry [32], given that industrial policies considerably influence investment decisions, thereby impacting the industry’s outcomes [33, 34]. In sum, a definitive relationship exists between policy influence and the evolution of the sports industry. **2.4.2 Economic development levels and sports industry**

The expansion of the sports industry is contingent upon economic development, which underpins consumer spending power and bolsters market vitality. A mature economy typically correlates with an upsurge in sports enterprises and industrial clustering [35]. The industry’s magnitude is a function of the aggregation of consumer bases. Furthermore, elevated per capita disposable income facilitates the proliferation of sports enterprises, thereby catalyzing the industry’s progression [32].

## 3. Materials and methods

### 3.1 FSQCA method

Qualitative Comparative Analysis (QCA) is a case-oriented methodology that views cases as integrative units of causal conditions, emphasizing complex causal interconnections between configurations and outcomes. It employs set relations to deduce causality, discerning necessary and sufficient conditions for specific outcomes, thus providing a structured and holistic means for dissecting complex causality. QCA has proven methodologically advantageous for examining complex phenomena characterized by heterogeneity, concomitant conditions, asymmetric associations, and equifinality among cases [36]. This robustness has facilitated its broad adoption in diverse disciplines such as management, sociology, and political science.

This study employs fuzzy-set Qualitative Comparative Analysis (fsQCA) in assessing the sports industry, predicated on the following rationales: (1) Prior research has predominantly concentrated on the influence of isolated factors within the sports industry, often overlooking the combined effects of multiple determinants. Furthermore, this study’s data comprise multi-dimensional continuous variables, for which fsQCA is adept at analyzing under complex precondition scenarios. (2) fsQCA is apt for investigating the simultaneous influence of various factors on the sports industry’s development, with the aim of delineating pathways for its enhancement. Consequently, the TOE (Technology, Organization, and Environment) framework is utilized to scrutinize the determinants impacting the sports industry, integrating these through the fsQCA methodology to distill pathways for industry improvement.

### 3.2 Samples and data

This study investigates the drivers of sports sector expansion across China’s provincial administrative divisions. Provincial governments are pivotal within the hierarchical state structure, fulfilling the role of executing national mandates and crafting region-specific policy measures. Moreover, the provincial tier offers an abundance of data for analysis.

Outcome measures reflect the sports industry’s progression in a given region, quantified by the aggregate output of local sports-related activities. These metrics gauge the scope and caliber of sports endeavors over a specified period. Such data are derived from annual assessments of sports development across provinces.

To accurately evaluate the intensity of research and development (R&D), it is essential to consider critical metrics indicative of regional technological innovation and climate. A primary metric is R&D expenditure, which serves as a proxy for assessing the capacity for technical innovation [37]. This assumes greater significance in appraising the R&D vigor of regional industrial clusters. The data for this analysis is sourced from the 2021 Provincial Statistical Yearbook. Furthermore, the regional digitization development index has been utilized to quantify the extent of network digitization, incorporating aspects such as digital investment, infrastructure, economic development, societal digitization, and e-government. This index data was obtained from China Economic Weekly. Organizational robustness, particularly in the sports industry, is contingent upon the quality of its human capital. Competent and adept personnel are fundamental to fostering innovation, propelling technological advancement, catalyzing reform, and elevating quality and productivity [38]. Data on these critical human resource metrics are documented in the statistical bulletins issued by provincial sports authorities. Policy documents are critical for discerning the strategic emphasis and initiatives of the Chinese government concerning the sports industry. The volume of policy papers reflects the industry’s significance to policymakers. The ramifications of policy execution permeate the local economic fabric, influencing industrial composition, sports-related consumption, and job creation. Data for this study was collated from the official online portals of provincial governments and sports departments. Per capita GDP was employed as an indicator of regional macroeconomic health [39]. Information was sourced from the 2021 Provincial Statistical Yearbooks, and estimates for absent data points were calculated using the sports industry’s growth trajectory. As shown in Table 1.

**Table 1:** Classification table of variables and indicators.

Variable categories	Variable names	Detailed indicators	Data resources
**Outcome variables**	Sports industry development level	Gross value of spots industry	Statistical Yearbook
**Technical conditions**	Digital development level	Digital development index	China Economic Weekly
	Technological innovation ability	R&D intensity (the proportion of R&D expenditure in GDP)	Provincial Statistic Yearbook
**Organization conditions**	Human resources	Number of sports employment personnel	Statistical Bulletin of Provincial Sports Bureau
**Environment conditions**	Policy instrument	Number of normative policy documents of sports industry	People’s Government, Sports Bureau website
	Economic development level	Per capita gross regional product	Provincial Statistical Yearbook

## 4. Empirical analysis and results

### 4.1 Calibration of data

The efficacy of fuzzy-set Qualitative Comparative Analysis (fsQCA) hinges on meticulous data calibration, which necessitates translating raw variable values into corresponding membership scores. Essential to this process is the establishment of three pivotal anchor points for each variable: full membership, maximum ambiguity (the crossover point), and non-membership.

In our study, we employ the direct calibration method for data transformation. Adhering to the calibration standards proposed by Fan et al., and considering case-specific circumstances, we define the full membership threshold for one outcome variable and five condition variables at 0.95, the crossover threshold at 0.5, and the non-membership threshold at 0.05 [40]. As shown in Table 2.

**Table 2:** Data calibration anchors.

	Variables	Fully affiliated	Intersection	Fully unaffiliated
**Outcome variables**	Sports industry development level	1355.605	329.245	4.3815
**Technical conditions**	Digital development level	0.76	0.25	0.108
	Technical innovation ability	3.64	1.695	0.5105
**Organization conditions**	Sports human resources	8.6	3.75	0.8
**Environment conditions**	Policy instrument	117	20.5	2.35
	Economic development level	155334.5	64923.5	48139

### 4.2 A One-variate analysis of necessity

The present study utilizes fuzzy-set Qualitative Comparative Analysis (fsQCA) software to evaluate the necessary conditions for the sports industry’s developmental levels, with findings summarized in Table 3. Analysis reveals that the consistency levels for all conditions under scrutiny fall below the critical value of 0.9, indicating that no individual condition is exclusively imperative for the sports industry’s growth. This implies that the industry’s expansion is likely the result of an interplay among multiple conditions rather than the dominance of any one. Future inquiries should, therefore, consider a diverse array of conditions to understand how their collective dynamics contribute to the sports industry’s evolution.

**Table 3:** Analysis of the necessary conditions.

Antecedent condition	Outcome variable	
	High-quality development	Not high-quality development
**High digital development level**	0.859752	0.787818
**Low digital development level**	0.522279	0.445205
**High technology innovation capability**	0.832725	0.786749
**Low technology innovation capability**	0.522279	0.433071
**High human resources**	0.886779	0.764484
**Low human resources**	0.470416	0.425926
**High policy instrument**	0.696859	0.664345
**Low policy instrument**	0.604821	0.497596
**High economic development level**	0.824032	0.768932
**Low economic development level**	0.539737	0.452508

### 4.3 Adequacy analysis of conditional configuration

To discern the multifaceted drivers of performance in the sports industry, this study employs a group state analysis that scrutinizes the efficacy of various conditions in concert. Leveraging set theory, the analysis probes the extent to which a combination of factors is instrumental for favorable outcomes, thus charting a viable route toward optimal performance. The evaluation of group states mandates a tailored approach to consistency assessment, diverging from the criteria utilized for necessary condition analysis. Prevailing literature reports varied consistency benchmarks, such as 0.76 or 0.8, contingent upon the size of the sample set. For small to medium-sized datasets, the frequency threshold is advised to be set at 1, whereas larger datasets necessitate a higher threshold. In this research, the consistency threshold is established at 0.80 with a frequency threshold of 1, to ensure robust analysis. The results are shown in Table 4.

**Table 4:** Con group analysis of sports industry development level.

	network-human resources-dominated	Technological innovation-human resource dominated	Comprehensive type
Condition configuration	Configuration1	Configuration2	Configuration3
**Digital development level**	●		●
**Technology innovation ability**	•	●	●
**Sports human resources**	●	●	●
**Policy instrument**		•	
**Economic development level**	•	●	●
**Consistency**	0.903	0.942	0.936
**Original coverage**	0.415	0.46	0.659
**Unique coverage**	0.089	0.02	0.219
**Consistency of solution**	0.902		
**Coverage of solution**	0.768		

Note: "●" and "●" represent this condition exists, "•" represents this condition does not exist, "●" represents the core condition, "●" represents the edge condition, black represents exists or does not exist.

**Fig 2:**
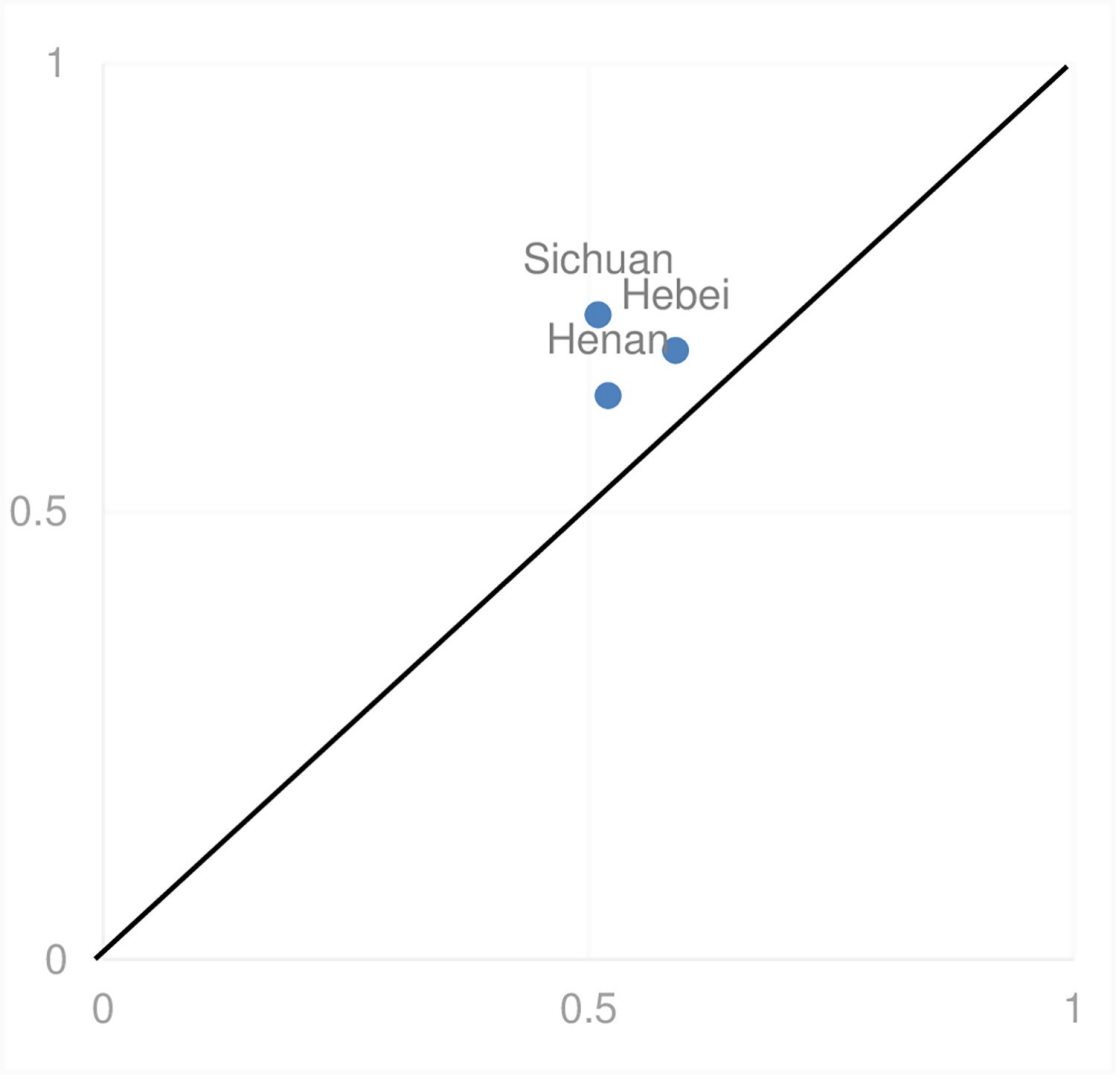
Interpretation case of configuration 1.

Table 4 delineates three group configurations that exhibit a consistency level surpassing the established minimum threshold of 0.75, both for individual group solutions and the aggregate outcome. The aggregate solution’s consistency is quantified at 0.902, indicating that these configurations are representative of well-developed sports industries in 90.2% of the investigated cases. Moreover, the coverage is reported at 0.768, suggesting that 76.8% of the cases with advanced sports industry development are accounted for by these groupings. The empirical analysis of this study is deemed valid as the consistency and coverage metrics both exceed their respective threshold values. Hence, the configurations identified in Table 4 are validated as a robust composite of conditions conducive to superior performance in the sports industry, with each configuration emphasizing distinct core conditions. Preliminary findings indicate that the synergy of digital development and sports-specific human capital is pivotal to the expansion of the sports industry. These elements can mitigate the absence of advanced industrial innovation and economic growth. In regions with a wealth of digital infrastructure and sports-related human resources, this amalgamation fosters the sports industry’s expansion, notwithstanding the shortfall in industrial and economic progress. Termed as the "network-human resources-dominated" model, this strategy is characterized by a high consistency index (0.903), considerable original coverage (0.415), and substantial unique coverage (0.816), accounting for 41.5% of the variations in sports industry development, with 8.9% uniquely ascribed to this model, as delineated in Fig 2.

**Fig 3:**
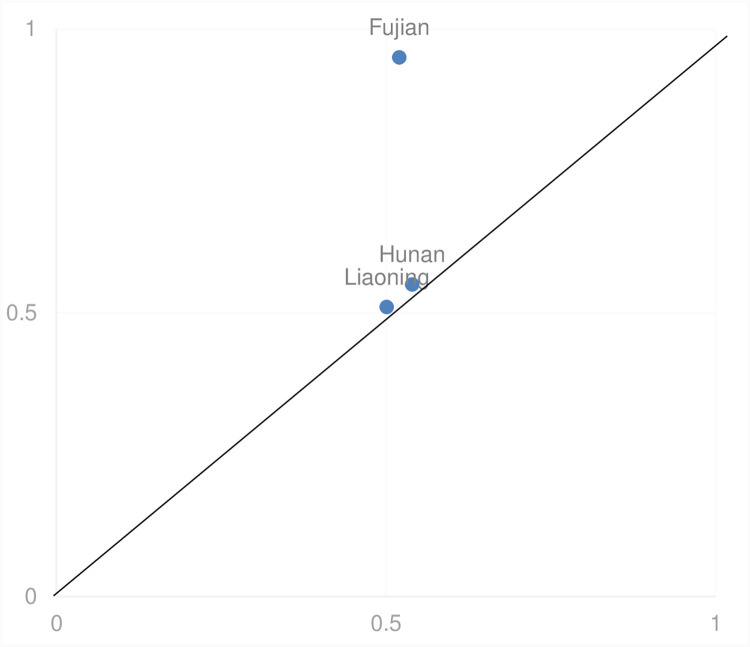
Interpretation case of configuration 2.

The second configuration demonstrates a high consistency rate (0.942), modest unique coverage (0.020), and substantial overall coverage (0.460), accounting for 46% of the variance in sports industry development, with an incremental 2% unique contribution (as depicted in Fig 3). In this model, technological innovation and human resources emerge as pivotal, whereas economic development assumes a supportive rather than a central role, with policy interventions being negligible. The data indicate that provinces can achieve significant advancements in the sports industry by leveraging innovation capabilities within the sector and optimizing human resource deployment, even in the absence of robust governmental support structures.

**Fig 4:**
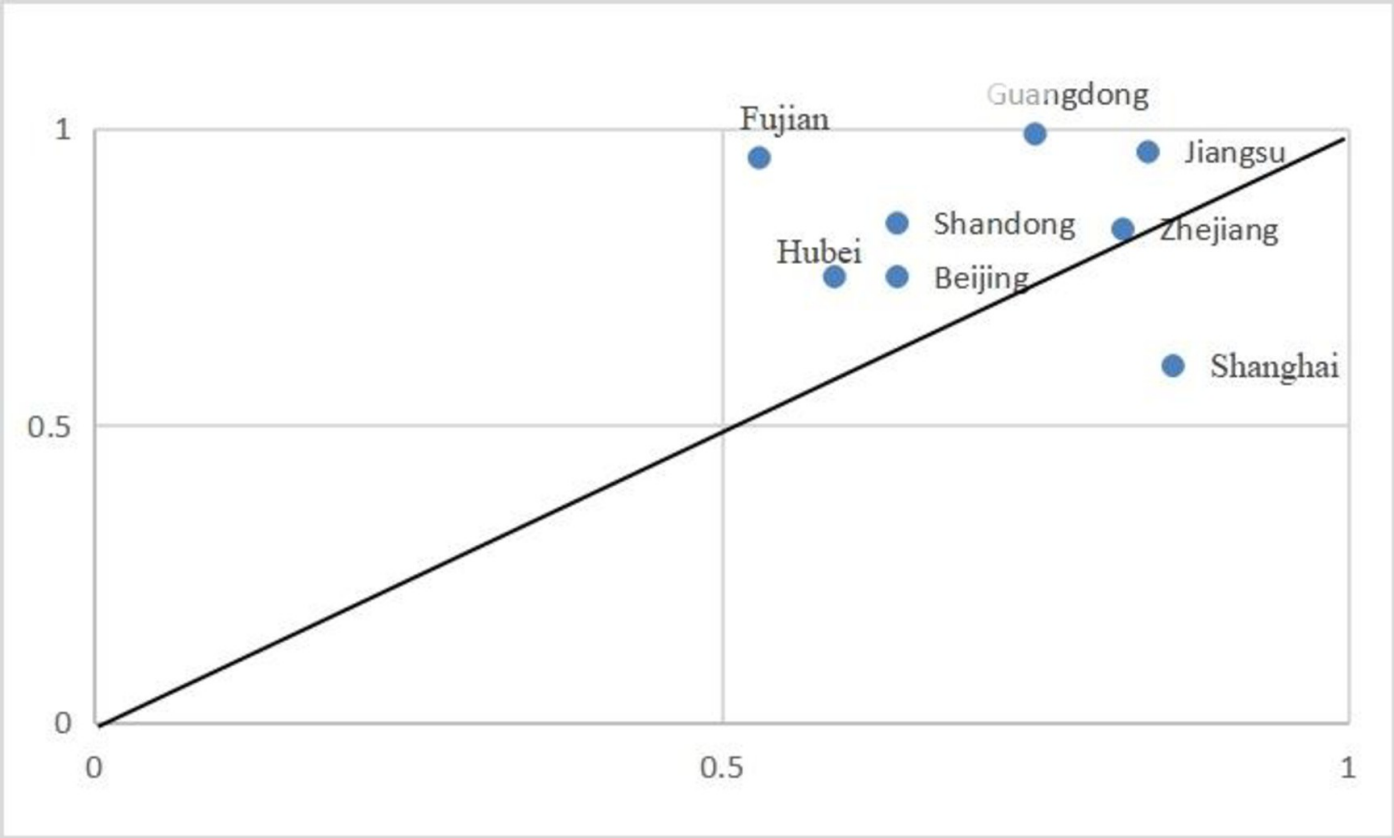
Interpretation case of configuration 3.

The third configuration highlights the pivotal role of digital development, innovation capabilities, and specialized human resources in fostering the sports industry’s growth. This model demonstrates robust internal consistency (0.936), with a substantial unique contribution (21.9%) to the variance in sports industry development—accounting for 65.9% of observed cases (refer to Fig 4).

### 4.4 Regional disparities in the development of china’s sports industry: East, central, and west

Provincial disparities in the digital sports industry in China are substantial, and influenced by geographic, resource-based, policy, innovative, human resource, policy instrument, and economic factors. Our research disaggregated the aggregate data into Eastern, Central, and Western regions to elucidate these variations. Comparative and analytical methods were applied to discern the influence of technological, organizational, and environmental factors on regional industry development, as detailed in Table 5.

**Table 5:** Analysis of sports industry development (high level) in east, middle, and west.

	East		Middle		West		
	Configuration 1	Configuration 2	Configuration 3	Configuration 4	Configuration 5	Configuration 6	Configuration 7
**Digital development level**	●	●	●	●	●	●	⊗
**Sports industry innovation ability**		●	●	●	●	⊗	⊗
**Human resources**	⊗	●		⊗	●	●	●
**Policy instrument**	⊗	●	●	⊗		●	⊗
**Economic development level**	●	●	●	⊗	●	●	⊗
**Consistency**	0.963	1	0.97	0.956	0.972	0.949	0.973
**Original coverage**	0.321	0.474	0.605	0.229	0.495	0.271	0.215
**Unique coverage**	0.101	0.253	0.501	0.125	0.381	0.149	0.075
**Consistency of solution**	0.979		0.975		0.955		
**Coverage of solution**	0.574		0.731		0.745		

Note: "●" and "●" represent this condition exists, "⊗" represents this condition does not exist, "●" represents the core condition, "●" represents the edge condition, black represents exists or does not exist

Table 5 presents two distinct strategic models for high-performance outcomes in the Eastern digital sports industry. The first model prioritizes digital and economic progress as pivotal drivers, designated as the "network-economy model," highlighting their role even in the absence of robust human resources and government focus. The second model, termed the "comprehensive linkage model," adopts a holistic approach, integrating digital progress, human capital, and policy instruments as fundamental components, with innovation capacity and economic growth serving as supportive elements.

as The central region’s sports industry achieves high performance through two principal configurations. Configuration 3 emphasizes digital development and sectoral innovation, supported by policy instruments and economic growth. Conversely, Configuration 4 underscores the importance of digitalization and innovation capacity within sports. Proximity to the technologically advanced eastern provinces allows the central region to leverage either configuration effectively.

The Western sports industry’s high performance is encapsulated by three strategic configurations. The "organization-environment type" (Configuration 5) centers on human resources and economic development, with digital development and industrial innovation serving as complementary factors. The "network-organization-environment type" (Configuration 6) emphasizes the interplay between digital development, human capital, and economic progress as foundational elements, supplemented by policy instruments. Configuration 7, the "organization type," singles out human resources as the pivotal factor.

## 5 Discussion

### 5.1 National-level analysis in China

#### 5.1.1 Network-human resources-dominated dual improvement path

This pathway underscores the primacy of digital development and strategic human resource management in sports, evidencing that digital progress is imperative for the sector’s high-quality growth and positioning sports human capital as critical for transforming the industry into a fundamental component of the national economy [41, 42].Provinces such as Sichuan, Hebei, and Henan serve as exemplars of this model. Sichuan, in particular, distinguished itself by topping the 2021 China Internet Development Index. To foster technological and economic growth, Sichuan launched the "Double Gigabit" Network Coordinated Development Plan in April 2021, aimed at enhancing network infrastructure, harmonizing applications, and integrating innovative services.

#### 5.1.2 Technological innovation-human resources leading dual improvement path

This strategy effectively addresses the shortfall in government support by harnessing the sports industry’s innovative capacities, human resource assets, and economic development levels, thereby facilitating superior progression in sports industry growth. The "technological innovation-human resources leading" model is demonstrated by Fujian, Hunan, and Liaoning, which rank in the mid-to-upper tiers of China’s provincial economic rankings, positioned at 8th, 9th, and 17th respectively in the 2021 China Digital Economy Development Report. Notably, Hunan’s "three highs and four news" initiative of 2021 aims to forge a niche in scientific and technological innovation and secure policy support to sustain this initiative. Moreover, on December 30, 2021, Hunan released a comprehensive plan to enhance the synergy between physical education and youth health initiatives. This plan outlines measures to improve compensation for sports educators, reinforce support for community sports programs in schools, streamline the recruitment of sports professionals, overhaul sports qualification frameworks, and promote the fluid movement and growth of talent in the sports sector.

#### 5.1.3 Technology-organization-environmen collaborative improvement path

This developmental path prioritizes technological and organizational imperatives, with environmental factors as supplementary conditions, and is predominantly observed in provinces with robust economic profiles. Provinces such as Beijing, Shanghai, Guangdong, Zhejiang, Shandong, Hubei, Fujian, and Jiangsu demonstrate this model, boasting not only abundant resource endowments but also leading China’s economic growth.

Shandong Province exemplifies this paradigm, achieving a commendable fourth rank in the World Internet Development Report 2021 and the China Internet Development Report 2021 for digital product innovation. Allocating 14.5 billion yuan to scientific research in 2022, the province is committed to technological progress. Through its "Ten Innovations" strategy, Shandong seeks to reinforce the interdependence of scientific research, technological projects, infrastructure, talent cultivation, and financial investment, thereby fostering high-quality development. Moreover, Shandong champions a "human-industry-urban" integration model, fostering collaborative dynamics among educational entities, government bodies, and enterprises to facilitate a reciprocal exchange of expertise and technology. This collaborative environment is further strengthened by financial incentives aimed at attracting and sustaining elite professionals and their cutting-edge contributions. The province also advances the "Shandong Elite Plan" and the "100 schools with 1,000 enterprises" initiative to engage online talent, incentivizing innovation and strategic human capital utilization.Such concerted efforts have significantly accelerated the advancement of Shandong’s sports industry, leveraging its innovative prowess, strategic human resource management, and economic insight.

### 5.2 Regional-level analysis in China

#### 5.2.1 Analysis of the sports industry improvement path in the Eastern region

In the Eastern region, the prerequisites for achieving distinction within the sports industry are multifaceted, encompassing advanced technological infrastructure, strategic human capital management, and a steadfast dedication to both environmental sustainability and economic progression. Comparative analysis of extant models reveals the imperative for a harmonious integration of both central and ancillary factors within the technical, organizational, and environmental spheres, to cultivate an industry milieu conducive to high performance. This necessitates an integrative developmental trajectory, wherein data analytics emerges as a pivotal element in informing strategic decision-making. Discrepancies in the allocation of technological, organizational, and environmental resources, as evidenced between the Northeastern and Southeastern regions, potentially elucidate the differential strategic orientations observed within these locales.

#### 5.2.2 Analysis of the sports industry improvement path in the Central region

In the sports industry of the Central region, empirical evidence suggests that a high level of performance is predominantly attributable to advancements in technology. This relationship posits that technological capabilities are instrumental in overcoming limitations posed by organizational structures and environmental constraints, thereby serving as an integral determinant of industrial success. The role of technological innovation extends beyond mere compensation for deficiencies; it fundamentally reconfigures industrial frameworks. By fostering an environment conducive to the synergistic interplay of production factors, technology not only refines the allocation of resources but also catalyzes an increase in both resource efficiency and total factor productivity. Consequently, this paradigmatic shift underpinned by technological progress propels a reorientation of industrial momentum and engenders a systematic enhancement in operational efficiency.

#### 5.2.3 Analysis of the sports industry improvement path in the western region

The Western region, however, confronts technological, organizational, and environmental challenges, potentially attributable to its developmental stage. Disparities with the central and eastern regions in these domains persist. To mitigate such challenges and bolster the industry’s growth, a tripartite approach is recommended. Primarily, enhancing the quality of human capital is essential. Concurrently, augmenting regional economic development in tandem with human resources should be pursued. Lastly, the advancement of digital products should synchronize with the aforementioned strategies to yield a comprehensive enhancement of the industry.

## 6 Conclusion and enlightenment

### 6.1 Conclusion

This study develops a nuanced TOE (Technology, Organization, Environment) configuration analysis framework tailored for assessing sports industry development. Utilizing the fsQCA (fuzzy-set Qualitative Comparative Analysis) methodology, the research systematically investigates the configurational impact of technological, organizational, and environmental dimensions on the level of development in the sports industry. Furthermore, it delineates the trajectory for enhancement within these domains. The empirical findings reveal the following:

1. At a comprehensive level, the presence of technological, organizational, and environmental factors alone does not suffice as essential prerequisites for the sports industry’s development, nor can any single factor independently impede its growth. Pertaining to China’s sports industry, empirical research delineates three key drivers for high-performance development. Firstly, the "Digital-Network and Human Resources-Led" pathway focuses on leveraging digital advancements and human capital within the sports sector. Secondly, the "Tech-Innovation and Human Resources-Led" pathway emphasizes the concurrent strengthening of the industry’s innovative infrastructure and human resources. Lastly, the "Integrated Synergy" approach assesses the collective impact of digital networks, innovation capacity, and human resources. Collectively, these pathways outline a multifaceted strategic approach to foster high-quality development in the sports industry, offering a foundational theoretical framework to guide policy-making and implementation.
2. At the regional development level, the sports industry in Eastern, Central, and Western China exhibits distinct divergences in its developmental drivers. The Eastern region, leveraging its economic and network strengths, primarily employs "Network-Economic" and "Integrated Synergy" approaches to achieve high performance. The Central region tends toward a "Technological" pathway. In contrast, the Western region encompasses a more complex set of pathways including "Organizational-Environmental," "Network-Organizational-Environmental," and "Organizational" types, reflecting the heterogeneity of conditions conducive to high-performance development in regional sports industries.

### 6.2 Enlightenment

The findings of this article offer the subsequent policy recommendations for practical development within the sports industry:

1. This study suggests on a holistic level, firstly, to promote the integration and innovative collaboration of the three key elements: technology, organization, and environment. Provincial departments should reinforce the competitiveness of their policies for sports talent. Secondly, to facilitate inter-provincial collaboration and resource sharing, with the aim of establishing a decentralized, networked development ecosystem.
2. Strategies for the development of the sports industry should be tailored to regional characteristics, with differentiated approaches. The eastern region should focus on enhancing the digitalization process, while the central region needs to consider both the level of data utilization and innovation within the sports industry. The western region should prioritize the improvement of sports human resources.

### 6.3 Limitations and prospects

This study has its limitations: Firstly, although it incorporates five key antecedents based on the research question for analysis, it may have overlooked potential factors that constitute core or necessary conditions for the development of the sports industry. Secondly, there is room for theoretical diversification. The current research is grounded in the TOE framework, and future studies could explore different theoretical perspectives to potentially yield novel insights into the development of the sports industry.Future research should focus on dynamically tracking and analyzing the developmental trends of the sports industry. By integrating the outcomes of case studies and comparing them with the findings of this paper, the universality of the conclusions can be strengthened.

## Supporting information

S1 File (ZIP)
